# Shape Alterations of Subcortical Nuclei Correlate With Amyotrophic Lateral Sclerosis Progression

**DOI:** 10.1002/brb3.70495

**Published:** 2025-05-19

**Authors:** Yanchun Yuan, Yan Fu, Xueying Wang, Fan Hu, Qianqian Zhao, Cailin He, Linxin Tang, Yongchao Li, Yue Bu, Xinyu Song, Qing Liu, Ziqin Liu, Renshi Xu, Wenfeng Cao, Yuanchao Zhang, Xiaoping Yi, Junling Wang, Bihong T. Chen

**Affiliations:** ^1^ Xiangya Hospital Central South University, Jiangxi (National Regional Center for Neurological Diseases) Nanchang Jiangxi P. R. China.; ^2^ National Clinical Research Center for Geriatric Diseases Xiangya Hospital, Central South University Changsha Hunan P. R. China; ^3^ Key Laboratory of Hunan Province for Neurodegenerative Disorders Central South University Changsha Hunan P. R. China; ^4^ Center for Medical Genetics, School of Life Sciences Central South University Changsha Hunan P. R. China; ^5^ Engineering Research Center of Hunan Province for Cognitive Impairment Disorders Central South University Changsha Hunan P. R. China; ^6^ Hunan International Scientific and Technological Cooperation Base of Neurodegenerative and Neurogenetic Diseases Changsha Hunan P. R. China; ^7^ Hunan Provincial University Key Laboratory of the Fundamental and Clinical Research on Neurodegenerative Diseases Changsha Hunan P. R. China; ^8^ Department of Radiology Xiangya Hospital, Central South University Changsha Hunan P.R. China; ^9^ Jiangxi Provincial People's Hospital, Clinical College of Nanchang Medical College First Affiliated Hospital of Nanchang Medical College Nanchang Jiangxi P.R. China; ^10^ Key Laboratory for NeuroInformation of Ministry of Education, School of Life Science and Technology University of Electronic Science and Technology of China Chengdu Sichuan P.R. China; ^11^ Department of Diagnostic Radiology City of Hope National Medical Center Duarte California USA

## Abstract

**Background:**

Neuroimaging has been increasingly used to assess brain structural alterations in patients with amyotrophic lateral sclerosis (ALS). We aimed to investigate alterations in brain sub‐cortical structures and to identify potential neuroimaging biomarkers for disease progression for patients with ALS.

**Methods:**

A total of 61 patients with ALS were prospectively enrolled and were divided into three subgroups according to disease progression, i.e., fast, intermediate, and slow progression. Sixty‐one matched healthy controls (HCs) were also recruited. All participants acquired a brain structural magnetic resonance imaging scan for subcortical volumetric and shape analyses. Neuropsychological testing and functional assessment were performed.

**Results:**

Patients with fast progression showed significant shape alterations in basal ganglia and brainstem as compared to the HCs group. In ALS patients with fast progression, shape contractions with atrophic changes were noted in bilateral nucleus accumbens, left caudate, left thalamus, and brainstem; while shape expansion with hypertrophy was noted in the left caudate, left thalamus, and left pallidum (all *p* < 0.05). There were significant positive correlations of the shape changes of the left thalamus with the Amyotrophic Lateral Sclerosis Functional Rating Scale‐Revised (ALS‐FRS‐R) total and limb scores and with disease duration (all *p* < 0.05). There were positive correlations of left pallidum with anxiety or with disease duration, and of left nucleus accumbens with ALS‐FRS‐R total or bulbar score, and of brainstem with mini‐mental state examination score (all *p* < 0.05).

**Conclusion:**

Extensive shape alterations of subcortical nuclei were noted in patients with fast progression of ALS, implicating subcortical shape being a potential neuroimaging biomarker for ALS progression.

## Graphical abstract

Extensive shape alterations of subcortical nuclei noted in amyotrophic lateral sclerosis (ALS) patients with fast progression implicated subcortical shape being a potential neuroimaging biomarker for ALS progression, facilitating early diagnosis and prompt management of patients with ALS.

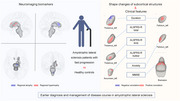

## 1 Introduction

Amyotrophic lateral sclerosis (ALS) is a progressive neurodegenerative disorder characterized by degeneration of upper and lower motor neurons, leading to muscular weakness and atrophy (Feldman et al. 2022). Patients with ALS present with a high degree of heterogeneity in clinical presentation, disease progression, and prognosis. For instance, a majority of patients with ALS die due to respiratory failure within three –five years of onset of symptoms (van Es et al. 2017), while approximately 10% of patients may survive longer than ten  years after diagnosis (Pupillo et al. 2014). The neuropathological basis for ALS progression remains unknown. Management is greatly affected by disease progression. Thus, it is prudent to identify the underlying neural correlates for ALS progression.

Advanced neuroimaging has been helpful to investigate the pathophysiological mechanisms in neurodegenerative diseases such as ALS (Castelnovo et al. 2023; Chiò et al. 2014; Mazón et al. 2018). Brain structural changes have been identified in patients with ALS, such as cortex thinning in the precentral and frontotemporal gyrus (Consonni et al. 2019), alterations of the corticospinal tract and corpus callosum on T2‐weighted images and diffusion‐tensor imaging (Fabes et al. 2017), and degeneration of motor and extramotor networks (Basaia et al. 2020). In addition, reduced volume and regional shape changes of subcortical structures such as the brainstem, thalamus, caudate nucleus, putamen, globus pallidus, nucleus accumbens, amygdala, and hippocampus have been reported in patients with ALS (Ahmed et al. 2021; Bede et al. 2013; Bede et al. 2016; Chang et al. 2005; Machts et al. 2015; Milella et al. 2022). Involvement of the hippocampus and basal ganglia has been included as important markers for staging and disease‐severity in a four‐stage pathological classification system of ALS (Brettschneider et al. 2013). Therefore, subcortical structures may hold important information regarding ALS progression.

Neuroimaging findings of subcortical structural changes have been reported to be correlated with disease progression in patients with ALS. A study by Tae et al. reported the regional shape contraction in the latero‐posterior surface of the right putamen, and the functional status of the patients with ALS was negatively correlated with local shape distance in the putamina (Tae et al. 2020). Prior studies have also investigated the volumetric changes of subcortical nuclei in patients with ALS. Agosta et al. found rapidly progressing patients with ALS had gray matter atrophy in the left caudate and right putamen as compared to the controls and non‐rapidly progressing patients using tensor‐based morphometry (Agosta et al. 2009). Senda et al. reported that the patients with rapidly progressed ALS presented more widespread and severe volume reduction in the basal ganglia, particularly in the head of the caudate and thalamus, as compared to patients with slow and intermediate progression (Senda et al. 2017). Nevertheless, there is no consensus as to how regional changes in the subcortical nuclei may affect the progression of ALS.

In this study, we prospectively enrolled patients with ALS and age‐ and sex‐matched healthy controls (HCs). Patients with ALS were divided into three subgroups according to their progression rate, i.e., fast, intermediate, and slow progression. All participants acquired structural brain magnetic resonance imaging (MRI) scans for volumetric and vertex‐based shape analyses of subcortical nuclei. We hypothesized that there would be different patterns of volume and shape alterations in subcortical nuclei in patients with different progression rates as compared with the HCs, and the changes of the subcortical nuclei were correlated with disease progression in patients with ALS.

## 2 Materials and Methods

### 2.1 Study Population

**FIGURE 1:**
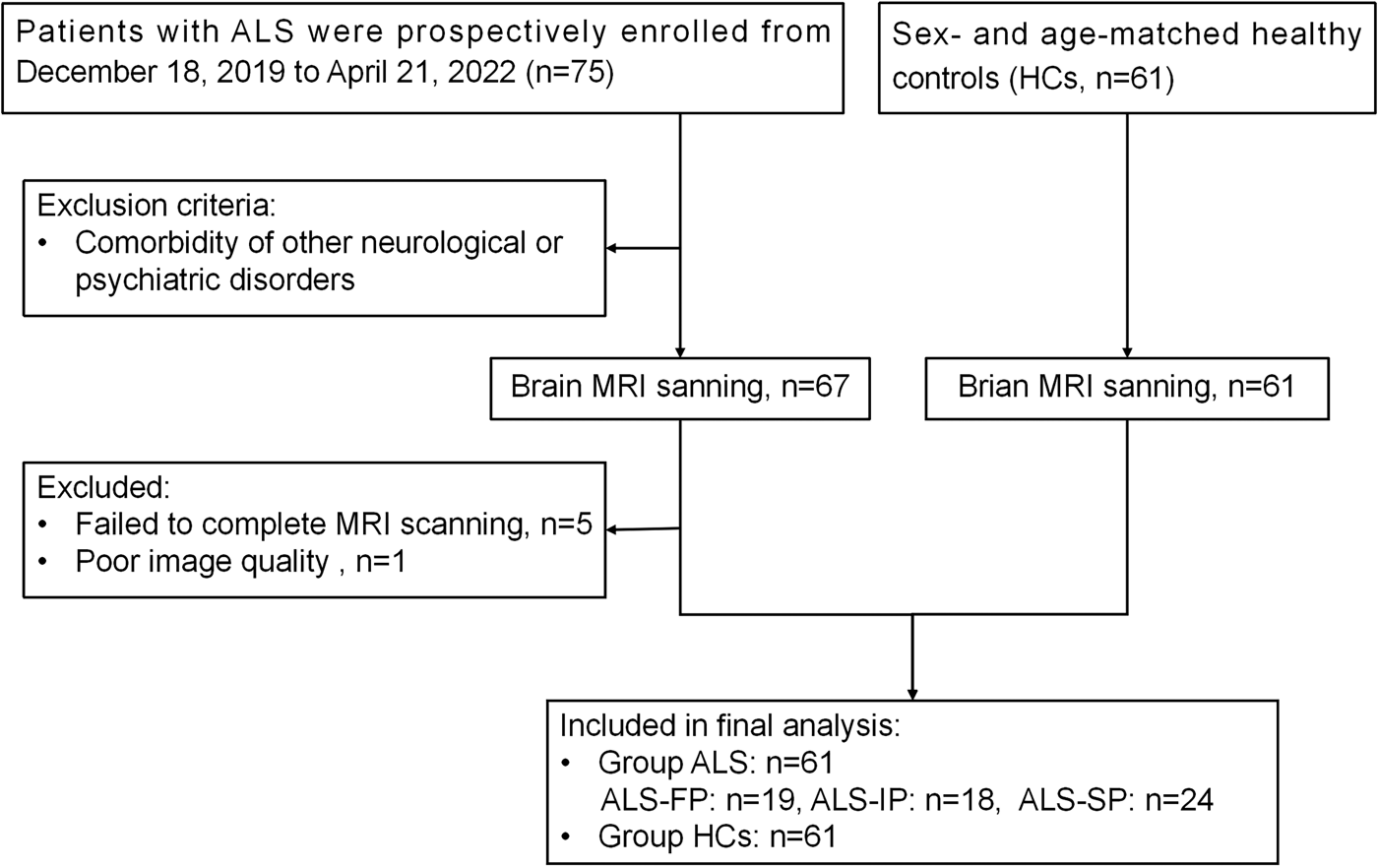
Flow chart illustrating the enrolment process. **Abbreviations**: ALS, amyotrophic lateral sclerosis; FP, fast progression; IP, intermediate progression; SP, slow progression.

Patients with ALS and age‐ and sex‐ matched HCs were prospectively enrolled into the study. Patients were recruited from the Neurology Clinics at Xiangya Hospital, Central South University, People's Republic of China and Xiangya Hospital, CSU, Jiangxi Hospital, National Regional Center for Neurological Diseases, People's Republic China from July 2021 to November 2022. All patients were diagnosed with ALS by at least two experienced neurologists according to the Gold Coast criteria for ALS (Shefner et al. 2020). The exclusion criteria for patients with ALS were as follows: (1) having comorbidities of other neurological or psychiatric disorders; and (2) being unable to complete the brain MRI scan due to physical or psychological factors. All the enrolled patients were sporadic, and the pathogenic mutations in known ALS‐causative genes were excluded by whole exome sequencing. The inclusion criteria for the HCs included the following: age‐ and sex‐ matched individual with no history of neurodegenerative disorders. The exclusion criteria for the HCs were as follows: (1) showing signs of neurological disease; (2) with cognitive impairment by medical history; and (3) with neuroimaging abnormalities such as stroke, tumor or dementia pattern on brain MRI. Figure 1 presents the details for study enrollment. This study was approved by the Ethics Committee and Institutional Review Board of Xiangya Hospital, Central South University, People's Republic China. Written informed consent was obtained from all participants.

Demographic and clinical data were obtained, including age at enrollment, sex, education, family history, age at onset, site of onset, handedness, and disease duration. The patients were assessed for their functional status using the Amyotrophic Lateral Sclerosis Functional Rating Scale‐Revised (ALS‐FRS‐R) scores. Cognitive testing was performed with the mini‐mental state examination (MMSE). Depression and anxiety were quantified using the Hamilton Depression Rating Scale (HDRS) and Hamilton Anxiety Rating Scale (HARS), respectively. The ALS‐FRS‐R was a 12‐item questionnaire used to assess progression of disability in patients with ALS, divided into three sub‐scores: bulbar (item No# 1–3), limb (item No# 4–9), and respiratory (item No# 10–12) (Cedarbaum et al. 1999). The higher the ALS‐FRS‐R score, the more preservation of function was indicated.

The rate of disease progression at enrollment was assessed using the delta ALS‐FRS‐R score (ΔFS), which was calculated as the following: ΔFS = (48‐ALS‐FRS‐R score at the time of diagnosis)/disease duration from onset to diagnosis in months) (Tae et al. 2020). Patients were divided into three subgroups according to the ΔFS: ΔFS < 0.5 for the slow progression subgroup (ALS‐SP); 0.5 ≤ ΔFS < 1 for the intermediate progression subgroup (ALS‐IP); ΔFS ≥1 for the fast progression subgroup (ALS‐FP) (Kimura et al. 2006). Accordingly, the HC group was also divided into three subgroups, matched with the three ALS subgroups.

Cognitive impairment was assessed with a combination of MMSE score and the education level according to the criteria proposed for the Chinese version of MMSE (Katzman et al. 1988), which stated the cutoff value of MMSE score being defined as 17 in illiterate individuals, 20 in individuals with elementary education, and 24 in individuals with middle school or higher education.

### 2.2 Structural MRI Data Acquisition

All brain MRI data were acquired using the same in‐house Siemens MAGNETOM Prisma 3.0 Tesla scanner, fitted with a 64‐channel, phased‐array receive‐only head coil. Head movement was minimized by the application of foam padding within a head coil. High‐Resolution T1‐weighted 3D MPRAGE (Magnetization Prepared—Rapid Gradient Echo) structural sequence was acquired in sagittal orientation using the following parameters: voxel size of 1.0 × 1.0 × 1.0 mm^3^, acquisition matrix 256 × 256 mm^2^, repetition time [TR] 2300 milliseconds (ms), echo time [TE] 3.0 ms, flip angle = 9°, and 208 slices with no gap. Additional imaging data, such as the T2‐weighted sequence and fluid attenuation inversion recovery (FLAIR) sequence, were obtained for evaluation of incidental brain abnormalities. None of the participants showed gross brain abnormalities.

### 2.3 Subcortical Volume Analysis

Volumetric analyses of subcortical structures were performed for the following structures: bilateral hippocampi, amygdalae, caudate nucleus, nucleus accumbens, putamen, pallidum, thalamus, and brainstem. The automated volumetric measures of brain structures implemented in FreeSurfer were used for calculating the volumes of these subcortical nuclei and total intracranial volume (TICV). A one‐way analysis of covariance was conducted to compare volumes of subcortical structures and TICV between the patients with ALS and the HCs. TICV was also compared between the ALS patient groups and the HC groups. Volumes of subcortical structures were included as dependent variables, and study group allocation as the categorical independent variable. TICV was used as covariates. A *p*‐value < 0.05 was considered significant.

### 2.4 Subcortical Shape Analysis

Shape analysis was performed in the same subcortical structures as the volume analysis. Shape analysis was processed using the integrated registration and segmentation tool (FIRST) algorithm from the Functional Magnetic Resonance Imaging of the Brain (FMRIB) Software Library (FSL) (Patenaude et al. 2011; Smith et al. 2004). Briefly, using a deformable mesh model, FSL‐FIRST parameterized volumetric labels of each subcortical structure as surface meshes, which were composed of a set of triangles, and the apex of adjacent triangles was called a vertex (Kim et al. 2013). Since each structure had a defined number of vertices, the corresponding vertices could be compared between individuals and groups. We matched each participant's surface mesh to the mean surface in the standard space to eliminate any potential pose discrepancies (rotation and/or translation) of each structure since each participant's surface mesh existed in native space. Between‐group vertex‐wise comparisons were performed for each subcortical structure with 5000 permutations, using a generalized linear model (GLM) in FSL with diagnosis, age, and sex as covariates. The results were corrected for multiple comparisons using threshold‐free cluster enhancement (TFCE) (Smith and Nichols 2009) to a family‐wise error (FWE) rate of *p* < 0.05.

Pearson's correlation coefficient was used to determine the correlation between shape alterations and clinical variables, including disease duration, ALS‐FRS‐R scores, HDRS scores, HARS scores, and MMSE scores. Multiple corrections using FWE correction, with a significance level set at 0.05 (p_FWE_ < 0.05).

### 2.5 Statistical Analysis

Statistical analysis was performed with SPSS Statistics version 25.0 (IBM Corp., Armonk, NY). Continuous normally distributed clinical data were presented as mean (SD) and were tested by Student's *t* test or one‐way ANOVA test, and continuous non‐normally distributed data were presented as median (IQR) and compared by the Mann Whitney *U* test or Kruskal Wallis test. Categorical variables were compared using the Pearson chi‐square test or Fisher's exact test. Statistical significance was defined at *p*< 0.05.

## 3 Results

### 3.1 Demographic and Clinical Features

A total of 61 sporadic ALS patients and 61 HCs were enrolled in the study. The details of the demographic and clinical characteristics of the participants are summarized in Table 1 and Table 2. As shown, patients with ALS and the HCs were matched for age and sex. The mean age at onset for patients with ALS was 53.95 ± 10.89 years, the mean disease duration at the time of enrollment was 15.15 ± 10.39 months, and the mean ALS‐FRS‐R score at the initial visit was 40.92 ± 5.30 scores. Nine patients with ALS showed cognitive impairment based on the results of the MMSE test, with a median score of 20. In the ALS‐FP group, the average score of ΔFS was 1.76 ± 1.16, while in the ALS‐IP and ALS‐SP groups, it was 0.62 ± 0.10 and 0.28 ± 0.13, respectively.

**TABLE 1:** Demographic and clinical features of patients with ALS and in this study.

	All patients with ALS	Fast progression subgroup	Intermediate progression subgroup	Slow progression subgroup	*p*‐value
N of participants	61	19	18	24	—
Age, years ± SD	54.85±10.96	57.42±11.55	55.72±9.17	52.17 ±11.54	0.2769
Sex, male/female	34/27	10/9	10/8	14/10	0.9324
Education, years ± SD	9.56 ± 4.12	7.89 ± 2.87	9.78 ± 3.83	10.71 ± 4.82	0.0931
Disease duration	15.15 ± 10.39	10.79 ± 7.60	13.94 ± 5.09	19.50 ± 13.44	0.0138
Site of onset, limb/bulbar	52/9	17/2	14/4	21/3	—
ALS‐FRS‐R					
Mean score ± SD	40.00±5.60	36.21 ± 6.98	40.33 ± 2.95	42.75 ± 4.15	0.0004
Bulbar	10.62±1.77	10.26 ± 2.10	10.11 ±1.97	11.29 ± 1.04	0.1088
Limb	17.95±4.43	15.21 ± 4.64	18.67 ± 3.38	19.58 ± 4.05	0.0044
Respiration	11.44±1.26	10.79 ±2.02	11.56 ± 0.62	11.88 ± 0.34	0.0648
ΔFS	0.84 ± 0.90	1.76 ± 1.16	0.62 ± 0.10	0.28 ± 0.13	< 0.0001
MMSE (median)	27	26	28	27	0.1865
HDRS (median)	4.5	4.0	7.0	3.0	0.0749
HARS (median)	6.0	5.0	9.5	4.0	0.1426

**Abbreviations**: ALS, amyotrophic lateral sclerosis; ALS‐FRS‐R, amyotrophic lateral sclerosis—functional rating scale‐revised scores; HARS, Hamilton anxiety rating scale. The comparation was performed between three subgroups. ΔFS, delta FRS‐R score; HDRS: Hamilton depression rating scale;. SD, standard deviation; MMSE, mini‐mental status exam.

**TABLE 2:** Characteristics of study participants including patients with ALS and the HCs.

	N	Age, years ± SD	Sex, male/female
Total ALS patients	61	54.85 ±10.96	34/27
Total HCs	61	54.79 ± 9.90	34/27
*p*‐value		0.8651	> 0.999
Fast progression ALS	19	57.42 ± 11.55	10/9
HCs for fast progression ALS	19	57.42 ± 10.13	10/9
*p*‐value		0.9250	> 0.999
Intermediate progression ALS	18	55.72 ± 9.17	10/8
HCs for intermediate progression ALS	18	55.44 ± 7.86	10/8
*p*‐value		0.9313	> 0.999
Slow progression ALS	24	52.17 ± 11.54	14/10
HCs for slow progression ALS	24	52.21 ± 10.81	14/10
*p*‐value		0.8740	> 0.999

***Note***: No significant differences were noted between the three HC subgroups (*p* > 0.05).

### 3.2 Volume and Vertex‐Based Shape Analysis

There was no significant difference in the volumes of all subcortical structures and TICV between the patients with ALS and the HCs (*p* > 0.05).

**FIGURE 2:**
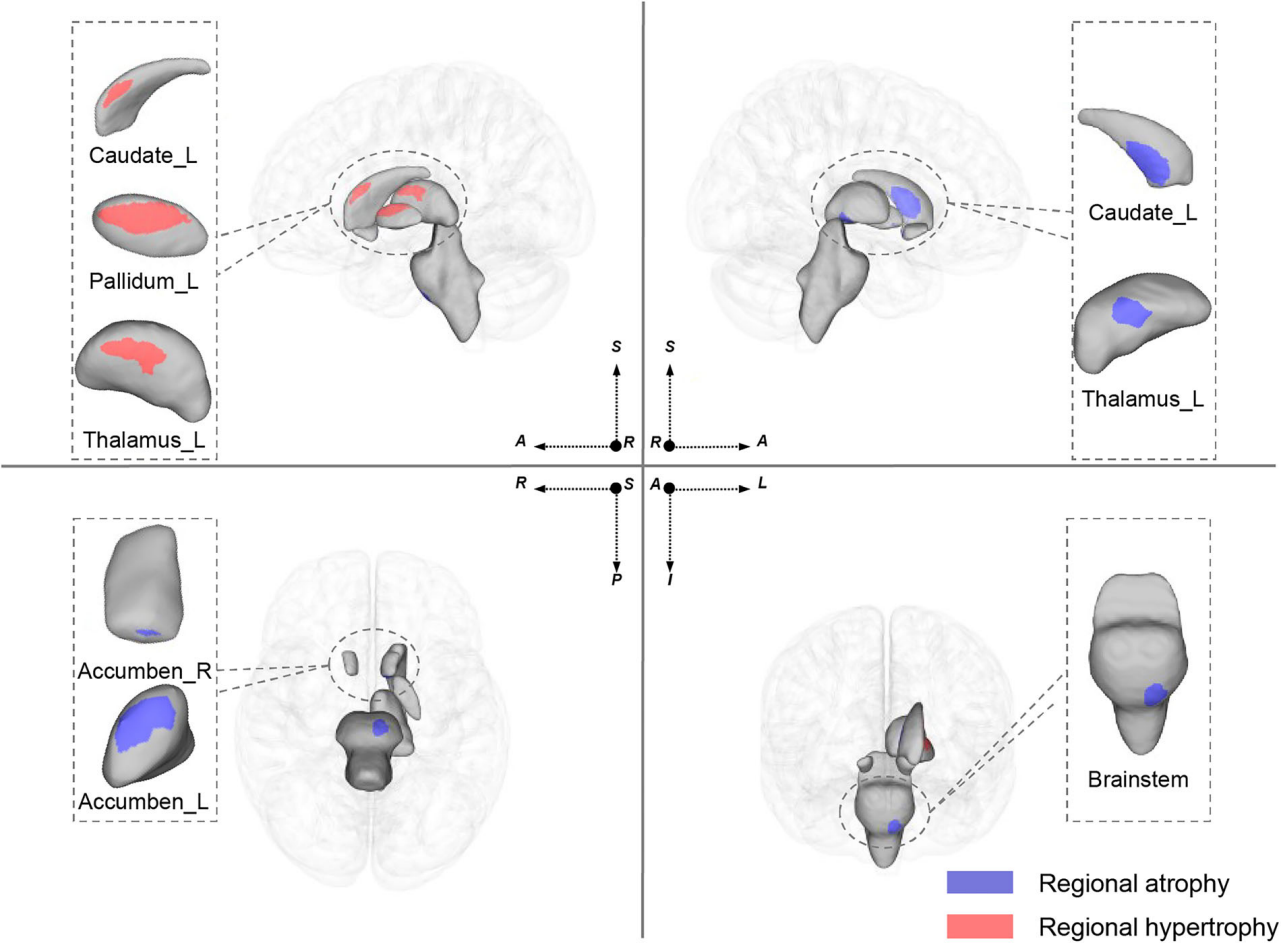
Significant vertex‐based shape differences between the patients with ALS with ALS‐FP and the HCs after multiple comparison correction to a Family‐Wise Error (FWE) rate of *p* < 0.05. **Abbreviations**: L, left; R, right.

A significant vertex‐based shape difference was observed between the ALS‐FP and the HCs after multiple comparisons with FWE (p_FWE_ < 0.05). Inward shape contraction with atrophy was noted in the bilateral nucleus accumbens, left caudate, left thalamus, and brainstem (p_FWE_ < 0.05) (Figure 2). Outward regional shape expansion with hypertrophy was noted in the left caudate, left thalamus, and left pallidum (p_FWE_ < 0.05) (Figure 2). Vertex‐Based shape analysis did not reveal a significant difference between the ALS‐SP and ALS‐IP groups as comparison to the HC group (p_FWE_ > 0.05).

### 3.3 Correlation Between Shape Changes and Clinical Features in the ALS‐FP Group

**FIGURE 3:**
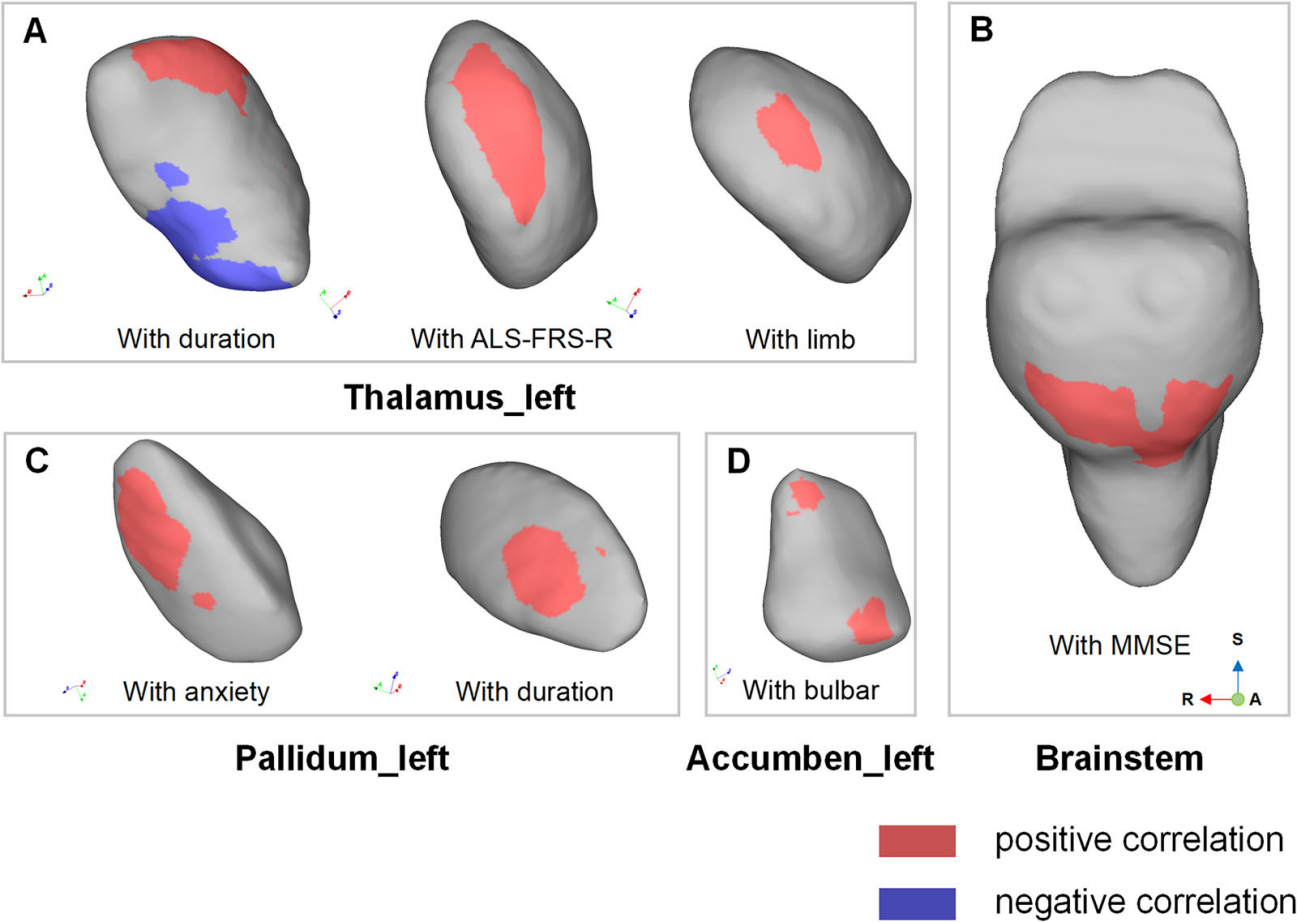
Significant correlation between shape changes and clinical features in the subgroup of patients with amyotrophic lateral sclerosis (ALS) and fast progression (ALS‐FP group). **(A)** Correlation of the shape changes in the left thalamus with disease duration, with ALS‐Functional Rating Scale‐R (ALS‐FRS‐R) and with ALS‐FRS‐R for limb. **(B)** Correlation of the shape changes in the brain stem with the mini‐mental status exam (MMSE) score., **(C)** Correlation of the shape changes in the left pallidum with anxiety score and disease duration., and **(D)** Correlation between the shape changes in the left nucleus accumbens and ALS‐FRS‐R bulbar score (all p_FWE_ < 0.05).

Pearson correlation analysis was performed to determine the association between the shape alterations and clinical features in the ALS‐FP group. The ALS‐FRS‐R score for disease severity was positively correlated with the shape alterations of the left thalamus and left nucleus accumbens (p_FWE_ < 0.05). The ALS‐FRS‐R limb score was positively correlated to the shape alterations of the left thalamus (p_FWE_ < 0.05), and the ALS‐FRS‐R bulbar score was positively correlated to the shape alterations of the left nucleus accumbens (p_FWE_ < 0.05). There was a significant positive correlation between disease duration and inward shape contractions in the left thalamus and left pallidum (p_FWE_ < 0.05). There was significant positive correlation between left pallidum and the anxiety score (p_FWE_ < 0.05). Positive correlation was also identified between the MMSE scores and brainstem shape contraction (p_FWE_ < 0.05). No significant correlation was identified between the subcortical shape changes and the ALS‐FRS‐R respiratory score or depression scores (p_FWE_ > 0.05). The correlation analysis data is presented in Figure 3.

## 4 Discussion

In this study, we identified significant shape alterations in the basal ganglia and brainstem of the ALS subgroup with fast progression, as compared to the other two ALS subgroups with no fast progression or the controls, which was correlated with disease duration, anxiety scores, ALS‐FRS‐R scores for disease severity, and MMSE scores for cognitive function. These results implicated the subcortical shape changes as being a potential neural correlate for the progression of disease in patients with ALS, providing new insight into the pathogenesis of ALS and potential regulation of disease progression.

Our findings identified shape alterations rather than volume changes of subcortical structures in patients with fast progression. These results were not entirely consistent with literature, which has shown significant subcortical volume changes in patients with ALS, and some but not all the volume changes have been associated with progression. For instance, regional volume change in basal ganglia was reported to be correlated with fast progression and shorter survival, and the main involved nuclei were the thalamus and caudate (Agosta et al. 2009; Westeneng et al. 2015). Gray matter atrophy and decreased fractional anisotropy were noted in the frontotemporal lobes and basal ganglia, including the thalamus and caudate, in patients with fast progression of ALS (Senda et al. 2017). However, a study by Menke et al. reported decreased volume of the thalamus and caudate in patients with ALS but found no significant correlation with progression (Menke et al. 2014).

In the present study, we found shape changes related to rapid progression in more subcortical structures, including the thalamus, caudate, nucleus accumbens, and brainstem than the number of involved subcortical structures with volume changes reported in the literature. Compared with volume loss, regional shape change measured by the local shape distance reflects regional atrophy and hypertrophy. Our study results indicated that the regional shape change may not be sufficient to cause total volume loss. Moreover, some nuclei showed both regional atrophy and hypertrophy, such as the thalamus and caudate, in this study. The co‐existence of atrophy and hypertrophy may lead to no significant volume change. On the other hand, volume loss of subcortical structures has been reported to be more predominant in ALS patients with cognitive/behavioral deficits (ALSci/bi) and in *C9orf72*‐related ALS patients than in ALS cognitively normal patients (Ahmed et al. 2021; Bede et al. 2013; Castelnovo et al. 2023; Machts et al. 2015). Christidi et al. disclosed shape alterations in the caudate and putamen in ALSci/bi and ALS cognitively normal patients and volume loss only in ALSci/bi patients (Christidi et al. 2018). Taken together, our study showed the novel finding, i.e., the shape changes of subcortical nuclei may be a more sensitive indicator for disease progression in patients with ALS than their volume loss.

In this study, we observed a significant association between subcortical structural alterations and clinical features in the subgroup of patients with fast progression. The basal ganglia structures are anatomically connected to the motor cortex, contributing to motor control, and are involved in the pathological changes in patients with ALS (Bede et al. 2013; Brettschneider et al. 2013; Castelnovo et al. 2023). In addition, the subcortical changes were reported to be related to extra‐motor cognitive and neurophysiological changes in patients with ALS (Bede et al. 2018; Machts et al. 2015), supporting our finding of a correlation with MMSE and anxiety scores. Abnormalities of the cortical‐basal ganglia network were found in patients with ALS (Basaia et al. 2020; Xu et al. 2017). Our finding of correlation with the ALS‐FRS‐R score was also consistent with the literature. A study by Tae et al. showed regional shape contractions in basal ganglia being negatively correlated with the changes in the ALS‐FRS‐R scores (Tae et al. 2020).

Our study contributed novel information regarding the thalamus and its association with disease progression. The thalamus has been reported to be involved in the pathogenesis of ALS. In the second stage of phosphorylated 43 kDa TAR DNA‐binding protein (pTDP‐43) for ALS, the large neurons of the thalamic nuclei projecting to the cerebral cortex have shown the pTDP‐43 aggregates (Brettschneider et al. 2013). Thalamic degeneration and alterations of thalamo‐cortical connectivity have been demonstrated in vivo by diffusion‐tensor imaging (Thivard et al. 2007), magnetic resonance spectroscopy (Sharma et al. 2011), functional MRI (Lulé et al. 2010), and positron emission tomography studies in patients with ALS (Turner et al. 2004). A recent cross‐sectional study reported thalamic atrophy in patients with ALS at the King's Stage 3 (Liu et al. 2021). Additionally, iron deposition in the thalamus was reported to be associated with disease severity in patients with ALS (Li et al. 2022). Volume change and shape alteration in the thalamus have also been reported to be associated with cognitive decline, disease progression, and survival time in ALS (Agosta et al. 2009; Senda et al. 2017; Westeneng et al. 2015). In the present study, we also observed both regional atrophy and hypertrophy in thalamus in ALS patients with fast progression, and the alterations in the thalamus were related to disease severity, which was consistent with previous studies.

The nucleus accumbens plays a key role in motivation, reward‐seeking, and the integration of cognitive and affective information (Floresco 2015; McGinty et al. 2013). It has widespread connections with the ventromedial prefrontal cortex, which may affect cognitive function. Prior studies have reported the contribution of the nucleus accumbens to neuropsychological deficits in patients with ALS (Bede et al. 2013; Machts et al. 2015). However, we found no association between the nucleus accumbens and cognitive or behavioral scores such as the MMSE and anxiety scores. It may be partially explained by the low frequency of patients with cognitive impairment in the ALS subgroup with fast progression in our study. Neuroimaging studies have reported involvement of the nucleus accumbens in patients with ALS (Bede et al. 2013; Tae et al. 2020), patients with *C9orf72*‐related ALS (Bede et al. 2013), patients with primary lateral sclerosis (Finegan et al. 2019), and patients with ALS‐FTD (frontotemporal dementia) (Bede et al. 2018; Machts et al. 2015). Nucleus accumbens was noted to be associated with the third pathological stage of the disease, displaying the heaviest burden of pathology within the striatum (Brettschneider et al. 2013).

Interestingly, we observed a novel significant association between nucleus accumbens and ALS‐FRS‐R bulbar score in the ALS subgroup with fast progression. The ALS‐FRS‐R bulbar score has been the most commonly utilized scale to assess global pharyngeal swallowing function in patients with ALS (Cedarbaum et al. 1999). Its association with the nucleus accumbens, as shown in the present study, implicated the potential neuroanatomical correlate of the bulbar function in patients with fast‐progressed ALS. To the best of our knowledge, this study presented the first evidence of the nucleus accumbens being associated with bulbar involvement in patients with ALS.

The dorsal pallidum, i.e., globus pallidus, is critical for motor control as the primary components of cortico‐subcortical circuits, and it has been shown to be involved in patients with ALS, especially in patients with ALSci /ALSbi (Bede et al. 2018; Canna et al. 2021; Machts et al. 2015; Tae et al. 2020). We found shape changes in the left pallidum being associated with anxiety scores in the ALS subgroup with fast progression. A prior study showed a reduction of dopaminergic transmission in the globus pallidus with increased anxiety‐like behavior (G et al. 2020). It has also been shown in animal studies that a knockdown of *CRFR1* mRNA expression in the mice globus pallidus elicited a significant increase in anxiety‐like behavior (Sztainberg et al. 2011). The role of the globus pallidus in the anxiety of patients with ALS requires more investigation.

We found shape changes in the brainstem being associated with the MMSE score in the patients with fast‐progressed ALS, which was consistent with prior studies in patients with ALS (Mioshi et al. 2013). Brainstem involvement in cognition has been a topic of intense research on neurodegenerative disease recently. First, the pathophysiological process of tau protein‐related Alzheimer's disease has been shown to start in the brainstem nuclei, which preceded the observable changes in the cortex and hippocampus (Braak and Del Tredici 2015). Brainstem volumetric abnormality was observed in preclinical and prodromal Alzheimer's disease (Dutt et al. 2020). Second, patients with injury in the brainstem had cognitive impairment, mainly involving the executive and attention function (D'Aes and Mariën 2015; Fu et al. 2017). In addition, increased brainstem metabolism was correlated with cognitive function in patients with Parkinson's disease (Blum et al. 2018). Here, we provided the novel initial evidence of brainstem shape changes being associated with cognitive functioning in patients with fast‐progressed ALS.

We did not identify any changes in the hippocampus nor any association between the hippocampus and clinical features in patients with ALS. This could partially be explained by a prior report of hippocampal pathology being associated with cognitive impairments in patients with ALS‐FTD (Neumann et al. 2006). Most of the patients with ALS in our cohort did not have significant cognitive impairment and did not have dementia from FTD, thus not showing hippocampal changes. In addition, literature has shown that the greatest burden of the pTDP‐43 lesions in the hippocampus was noted in the last stage of the disease (stage 4) according to the neuropathologic staging system for ALS (Brettschneider et al. 2013). Our study cohort with a mean disease duration of ten months in the ALS patients with fast progression was not at the last stage of the disease, which may account for the lack of hippocampal changes in the present study.

There were several limitations to the study. First, the sample size was relatively small, which did not allow for additional analysis with stratification of covariates, including cervical spondylosis, underlying comorbidities such as hypertension, etc. Future studies with a large sample size and sufficient statistical power are needed to validate the results from this study. Second, this was a cross‐sectional study without follow‐up neuroimaging data to assess brain structural changes and their correlations with clinical outcomes over time in patients with ALS. Longitudinal studies will be needed to tease out the relevant neuroimaging biomarkers for ALS progression. Third, this study was limited with only the MMSE available for evaluating cognitive status in patients with ALS, which was not adequate. more detailed cognitive, behavioral, and clinical evaluations of the ALS sample were required in the future. Fourth, this study was limited as the HCs did not undergo cognitive or behavioral tests, which may hinder the comparability between the patient groups and the HC groups. Nevertheless, the inclusion criteria for the HCs were stringent, and a detailed medical history was obtained to ensure no history of cognitive impairment in the HCs. Last, there may be a selection bias in our study as the patients with fast progression may have less diagnostic delay due to disease severity.

In summary, we identified subcortical shape changes and their association with disease progression in patients with ALS. These brain structural alterations may serve as potential neuroimaging biomarkers for ALS progression, facilitating early diagnosis and prompt management of patients with ALS.

## Author Contributions

**Yanchun Yuan**: writing–original draft, writing–review and editing, and investigation. **Yan Fu**: writing–original draft, writing–review and editing, and investigation. **Xueying Wang**: Investigation. **Fan Hu**: writing–review and editing. **Qianqian Zhao**: investigation. **Linxin Tang**: investigation. **Yongchao Li**: investigation. **Yue Bu**: investigation. **Xinyu Song**: writing–original draft and investigation. **Qing Liu**: investigation. **Ziqin Liu**: investigation. **Renshi Xu**: investigation. **Wenfeng Cao**: investigation. **Yuanchao Zhang**: formal analysis, writing–review and editing. **Xiaoping Yi**: conceptualization, writing–review and editing, formal analysis and funding acquisition. **Junling Wang**: conceptualization, supervision, writing–review and editing and funding acquisition. **Bihong T. Chen**: supervision.

## Conflicts of Interest

The authors declare no conflicts of interest.

## Ethics Statement

This research was approved by the Ethics Committee and Institutional Review Board in our hospital (IRB No. 202103191).

## Consent

Written informed consent was obtained from all participants.

### Peer Review

The peer review history for this article is available at https://publons.com/publon/10.1002/brb3.70495

## Data Availability

The data that support the findings of this study are available from the corresponding author upon reasonable request. Details about the datasets would be available from the corresponding authors (J.W., X.Y., and Y.Z.) upon reasonable request.
